# A randomised controlled trial of a theory-based intervention to improve sun protective behaviour in adolescents ('you can still be *HOT *in the shade'): study protocol

**DOI:** 10.1186/1471-2407-12-1

**Published:** 2012-01-03

**Authors:** Anna L Hawkes, Kyra Hamilton, Katherine M White, Ross McD Young

**Affiliations:** 1 Viertel Centre for Research in Cancer Control, Cancer Council Queensland, 553 Gregory Terrace, Fortitude Valley, Brisbane 4006, Australia; 2 School of Public Health, Queensland University of Technology, Victoria Park Road, Kelvin Grove 4059, Australia; 3 School of Psychology and Counselling, Queensland University of Technology, Victoria Park Road, Kelvin Grove 4059, Australia; 4 Institute of Health and Biomedical Innovation, Queensland University of Technology, 60 Musk Avenue, Kelvin Grove 4059, Australia

**Keywords:** Oncology, Skin cancer, Adolescent, School, Intervention, Theory of planned, Behaviour, Education, Sun protective behaviour

## Abstract

**Background:**

Most skin cancers are preventable by encouraging consistent use of sun protective behaviour. In Australia, adolescents have high levels of knowledge and awareness of the risks of skin cancer but exhibit significantly lower sun protection behaviours than adults. There is limited research aimed at understanding why people do or do not engage in sun protective behaviour, and an associated absence of theory-based interventions to improve sun safe behaviour. This paper presents the study protocol for a school-based intervention which aims to improve the sun safe behaviour of adolescents.

**Methods/design:**

Approximately 400 adolescents (aged 12-17 years) will be recruited through Queensland, Australia public and private schools and randomized to the intervention (n = 200) or 'wait-list' control group (n = 200). The intervention focuses on encouraging supportive sun protective attitudes and beliefs, fostering perceptions of normative support for sun protection behaviour, and increasing perceptions of control/self-efficacy over using sun protection. It will be delivered during three × one hour sessions over a three week period from a trained facilitator during class time. Data will be collected one week pre-intervention (Time 1), and at one week (Time 2) and four weeks (Time 3) post-intervention. Primary outcomes are intentions to sun protect and sun protection behaviour. Secondary outcomes include attitudes toward performing sun protective behaviours (i.e., attitudes), perceptions of normative support to sun protect (i.e., subjective norms, group norms, and image norms), and perceived control over performing sun protective behaviours (i.e., perceived behavioural control).

**Discussion:**

The study will provide valuable information about the effectiveness of the intervention in improving the sun protective behaviour of adolescents.

## Background

Skin cancer is the most prevalent form of cancer in Australia, accounting for approximately 80% of all new cancers diagnosed annually [1,2]. For Australians, this translates into 380,000 treated cases of skin cancer per year [1,2]. The numbers of new cases of skin and lip cancers (excluding non-melanoma skin cancers) for Australian women are projected to increase by 27% in the year 2011 [1]. For Australian men, projected increases in skin and lip cancer cases are even higher at 32% [1]. Exposure of the skin to the sun is the most consistently implicated factor causing skin cancer, and is an important concern for Australians, particularly in Queensland, which has the highest incidence rates of skin cancer and mortality rates for malignant melanoma in the world [3]. Most skin cancers are preventable by encouraging consistent use of sun protection methods including using a broad spectrum water resistant sun protection factor (SPF) 30+ sunscreen, staying in shady areas, minimizing time in the sun between 10 am and 3 pm, and wearing a wide brimmed hat, sunglasses, and protective clothing to reduce sun exposure and sunburn [4]. Since sun protective behaviour depends on individual decision making processes, it is vital to understand people's attitudes toward, and motivations for, sun protection.

While previous research has focused on raising awareness and knowledge about the dangers of skin cancer and measuring the adoption of sun safe practices, there is little research aimed at understanding why people do or do not engage in sun protective behaviour [5]. International research indicates that the choice to use sun protection is likely to involve psychosocial factors such as attitudes, normative influences, and efficacy [6]; however, few studies have focused on understanding the psychosocial processes surrounding sun protection in an Australian context, with an associated absence of theory-based interventions.

In general, there is high awareness and knowledge about skin cancer risk in the community, and people's attitudes are fairly positive about performing sun protection [7]. However, these factors do not necessarily translate into attitudinally-consistent behaviour. The decision to use sun protection is complex, involving a range of situational and motivational factors. In particular, adolescents' sun safe behaviours may depend on the context of the situation where notable increased compliance to sun protect occurs in the school context [8], and are likely to be motivated by referent group norms (e.g., peer and friendship groups; [9]) and image norms disseminated by the media (e.g., perceptions of a tan as attractive; [10,11]). Furthermore, there is individual variation in the types of sun protection used and the frequency and adequacy of their use [5]. Specifically, adolescents have high levels of knowledge and awareness of the risks of skin cancer but engage in few sun protective behaviours [12], and have been reported to intentionally use a low SPF sunscreen or deliberately expose themselves to the sun to obtain a tan [7]. This deliberate exposure to the sun to obtain a tan is supported by more recent adolescent sun safety research which suggests having a strong desire for a tan is associated with delaying the use of and using no sun protection [13]. The lack of correspondence between attitudes and behaviour has long been a focus for social psychology with many researchers arguing that a focus on people's attitudes do not account for the range of influences that may potentially guide behaviour [14].

**Figure 1:**
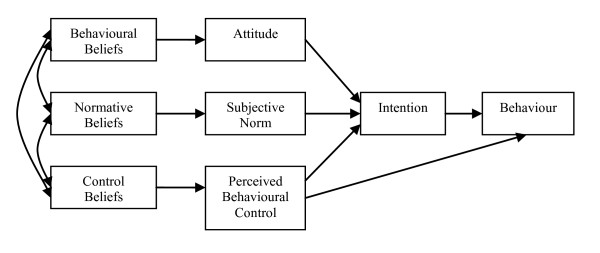
**The Theory of Planned Behavior **[15].

The Theory of Planned Behaviour [TPB; 15] is a model developed in response to identified inconsistencies between people's attitudes and actions (see Figure 1). The TPB is a well-validated model that has been used to explicate the attitude-behaviour relationship and accounts for the complexity of people's decision making. It specifies intentions as the most proximal determinant of behaviour with intentions being influenced by attitude (positive or negative evaluations of performing a behaviour), subjective norm (perceived social pressure to perform or not perform a behaviour), and perceived behavioural control (perceived ease or difficulty of performing a behaviour; also thought to be a direct predictor of behaviour; [15]). Attitude, subjective norm, and perceived behavioural control are informed by underlying behavioural (i.e., costs and benefits), normative (i.e., specific referents' (dis)approval), and control beliefs (e.g., barriers and facilitators), respectively, and it is these beliefs that can be used to design interventions [16]. According to the theory, other factors relevant to sun protection decisions, such as sun safety knowledge or perceptions of risk for skin cancer/damage, are not believed to influence intentions or behaviour directly but would instead be expected to inform underlying sun protection beliefs [15].

Support for the TPB has been demonstrated in several meta-analyses including Armitage and Conner's [17] study which found, across a range of social and health behaviours, that the model accounted for an average of 39% and 27% of the variance in intentions and behaviour respectively. The TPB constructs have been used successfully by several international researchers [6,10,11] and a smaller number of Australian researchers [9,18,19] to understand the motivations underlying sun protection-related behaviours. The results of these studies demonstrate that the TPB is a useful theoretical framework for examining the prediction of sun protective practices.

Despite support for the TPB, the normative component of the model, which reflects social pressure from significant others to perform the behaviour [15], has emerged consistently as the weakest predictor of sun protective intentions [6,19], a trend which concurs with previous meta-analytic research [17]. Researchers, drawing on social identity theory [20] and self-categorization theory perspectives [21] have advocated for a re-conceptualization of the normative component in the TPB to consider the influence of the expectations and actions of a specific, salient, reference group (i.e., group norms) on intentions and behaviour [19,22].

Group norms involve a consideration of whether important group members perform the behaviour (i.e. behavioural norm) and the evaluation of the behaviour by the group (i.e. group attitude). In the case of sun protection, for instance, people may be more likely to use sun protection if they believe that it is a usual and valued behaviour performed by other group members (e.g., friendship groups for adolescents). Terry and Hogg [19] found support for the positive effect of group norms (rather than subjective norms), in an extended TPB, on Australian female university students' intentions to sun protect. Similarly, White et al. [9] found a direct effect for group norms (in addition to subjective norms) on young Australians' sun protection intentions and behaviour.

Image norms are also another potential source of normative influence relevant to people's intentions and behaviour to sun protect [10]. For example, many adolescent females deliberately expose themselves to the sun with the sole purpose of developing a tan because a person with a tan is perceived as more attractive and healthy. Image norms reflect the self-presentational concerns of individuals about their image and are the cognitive representations of stereotypical members of particular groups (e.g., tanned people) [10]. Image norms are more distant from the individual than immediate referent norms (i.e., subjective or group norms) and are a general representation of the values of society as a whole (e.g., the media). Jackson and Aiken [11] focused on changing normative perceptions about the attractiveness of being tanned. They suggested that increasing the attractiveness of pale image norms may be effective in producing sun protective behavioural change [11].

Given the potential importance of social influences on adolescent sun protection decision making, it is important for researchers to consider targeting a range of different sources of social influence when developing programs to improve adolescent sun safe practices. In consideration of the useful contribution of group and image norms, within models such as the TPB, to predicting sun protection it seems warranted that these sources of social influence form a key focus of sun safety intervention programs.

Most sun safety interventions are educational in nature, designed to increase awareness and sun safety knowledge or perceptions of risk for skin cancer/damage [23]. While it is important to promote awareness about the effective use of sun protection, increasing people's knowledge and awareness of risk has not been shown to increase sun protection behaviour and there is a recognized need, both in Australia and internationally, for more novel interventions targeted at both adults and adolescents [23,24]. The belief basis of the TPB is useful in developing interventions to encourage behavioural change and may involve altering existing behavioural, normative, and control beliefs or exposing participants to new beliefs [16]. Hardemann et al. [25] reviewed 24 TPB intervention studies (21 of which were health related) and concluded that approximately half of the interventions were successful in changing intentions, with two-thirds successful in changing behaviour.

Two US-based interventions [11,26] have used some, but not all, components of the TPB to engender sun protection behaviour change. Mahler et al. [26] found that primarily female university students exposed to UV photo and photoaging stimuli who also received supportive information related to two types of norms (personal norms - what one 'should' do, and descriptive or behavioural norms about the sun protection behaviour of friends and peers) showed greater levels of self-reported sun protection behaviour than control participants over a 1-month period. The study focused on risk-related factors (susceptibility to a decline in health and appearance as a result of sun exposure). According to a TPB perspective, however, any risk-related factors would be reflected in the underlying costs (attitudes) and control perceptions. As Mahler et al.'s [26] study did not include a consideration of norms in the context of other known influences on sun safe behaviour such as attitudinal and control factors, it is not possible to determine the effects of these norms within the context of a comprehensive model of decision making.

Using appearance-based stimuli, Jackson and Aiken's [11] study of female university students' sun protection behaviour showed that, relative to the control group, the intervention increased participants' immediate perceptions of the benefits of sun protection, efficacy for sun protection, and image norms for being pale, as well as sun protection intentions with increases in intentions and behaviour at a 2-week follow-up. Although Jackson and Aiken [11] incorporated additional norms within the TPB, the original normative component of the model (i.e., subjective norm) was not included in the study, thus preventing a full comparison of the different sources of normative influence including the original conceptualization of norms proposed by Ajzen [15]. In addition, the generalisability of the findings of these two studies is limited by the focus on a single population (primarily female university students). Skin cancer rates in Australia are projected to increase more for men than women [1] and, adolescents, despite high levels of knowledge about the dangers of skin exposure to the sun, practice few sun protection behaviours [12]. Normative influences are especially salient for young people's health behaviour decision making [27], including their sun safe behaviours [9]. Thus, an assessment of the effectiveness of an intervention incorporating norms with a broader sample of respondents (e.g., males, adolescents) is particularly important.

The authors completed a pilot study [28] targeting the sun protection intentions and behaviours of young Queensland secondary school students (n = 80; 14.53 ± 0.69 years). Approximately half of the participants (n = 34) were exposed to the intervention with the other set of participants (n = 46) comprising a wait-list control group. The results revealed that students completing the intervention reported stronger sun-safe normative and motivator beliefs and intentions and the performance of more sun-safe behaviours across time than those in the control condition. However, while the results of the pilot intervention evidenced some positive changes in high school students' sun protection intentions and behaviour, the mechanism by which these changes occurred was unclear due to the limited number of participants providing follow-up data and the short follow-up time frame. Therefore, there is a need for refinement and replication of the intervention and evaluation of its components with a larger sample of participants. The present study builds on this successful pilot work to conduct a large-scale trial of this approach.

This paper presents the study protocol for a large-scale school-based intervention to improve sun protective behaviour in adolescents. The research will use an extended version of the TPB [15] to develop and test the utility of a sun protective intervention derived from this approach. The intervention will target previously identified costs and benefits, important referents, and barriers and motivators. We hypothesise that adolescents exposed to the intervention will report a significant improvement in their beliefs, intentions, and behaviour for sun safety from pre- to post-intervention compared to adolescents in the control group. We expect a significant improvement over time for all constructs, except for control belief barriers where a decrease is expected. This research will address a gap in the literature given the paucity of interventional sun safety research in Australian, and the results of this study will provide valuable new information about an intervention to improve sun protective behaviour in adolescents where timely strategies are required to develop lifelong sun protection habits.

## Methods/design

### Study design

The study is a two-armed prospective randomised controlled trial in which approximately 400 male and female adolescents aged 12 to 17 years will be randomised in a 1:1 ratio to the intervention or a wait-listed control group using a computer-generated random number sequence. Participants in both groups will complete assessments at baseline and post-intervention.

### Study Aim

The aim of this study is to evaluate the effectiveness of a TPB-based sun safety intervention for Queensland adolescents on increasing positive attitudes, normative support, and self-efficacy, leading to increased sun protection intentions and behaviour.

### Study Sample

#### Sample Eligibility Criteria and Recruitment Procedures

Ethics approval was received from the Queensland University of Technology Human Research Ethics Committee (approval number 1100000768). Eligibility criteria will include male and female students aged 12 to 17 years from public and private secondary schools across metropolitan and regional areas of Queensland, Australia.

A convenience sample of schools will be recruited using a maximum variation sampling method to ensure participating schools range in social-demographic status and geographical location. Schools will be approached to participate by the study team using an information package by phone, email and face-to-face discussions. The information package will include a letter to the school principal, background information about the study, questionnaire items, and the participant intervention workbook. Consenting schools will be requested to identify teachers (and their students) that will be accessible to the research team. Active consent will be obtained from both the student participating and their legal guardian. Baseline data will be collected from consenting students by the study team at a time and day specified by the school principal.

#### Sample size

A total of 400 participants (200/group) is aimed to be recruited. Based on our previous research in the area [28], it is anticipated that there will be approximately 35% attrition over 4 weeks of follow-up for reasons such as school absence or failure to complete follow-up questionnaires. A total sample of approximately 260 (400-140) completing participants (130/group) is required to detect a medium effect in sun safety behaviour. This sample size was determined by power analysis using the G*Power program [29,30]. Significance level (alpha) was established at 0.05 to avoid a Type 1 error, power (1-beta) was set at 95% to avoid a Type II error, and effect size was determined at .25. Therefore, for a 95% chance of detecting as significant a 4 week difference in sun safe behaviour, approximately 130 participants in each group are needed to complete the study.

### Study Conditions

#### Control

Control participants will be wait-listed to receive the study intervention. At the completion of the study, participating schools will be offered the opportunity to have trained facilitators run the program sessions to control group participants.

#### Intervention

The intervention will focus on: (i) encouraging supportive sun protective attitudes and beliefs, (ii) fostering perceptions of normative support for sun protection behaviour, and (iii) increasing perceptions of control/self-efficacy over using sun protection. Intervention sessions will be facilitated by trained persons from the Cancer Council Queensland and Queensland University of Technology. Facilitators and Queensland University of Technology research staff will be authorised to deliver the intervention with secondary school students during school hours, and all study staff directly involved with the participants and the running of the intervention will be approved to work with minors (have a current Positive Notice Blue Card). The facilitators and Queensland University of Technology research staff will follow the directions of supervising school staff regarding access to child participants and the location of testing. Testing will take place in an accessible area designated by the school principal or supervising teacher, in proximity to normal classroom activities. As required by the Health and Safety requirements for Queensland schools, the supervising teacher will be aware of the intervention testing circumstances. Assistance will be available from school staff when required. The facilitators and Queensland University of Technology research staff will follow the required procedures for visitors to the school.

The intervention will be delivered during three × one hour sessions over a three week period, and each session will address a different construct. Week one will be designed to encourage supportive sun protection related attitudes and beliefs. Week two will focus on fostering perceptions of friendship group normative support for sun protection. Week three will aim to increase perceptions of control/self-efficacy over using sun protection. Activities for the intervention will include group based discussions, practical sessions on being sun safe (e.g., role playing activities to convince friends to be sun smart), watching relevant sun safety DVDs, setting sun safe goals, and students creating their own internet and/or text message campaigns to encourage sun safety amongst young people. At the conclusion of each session, participants and facilitators will evaluate program content, materials, and delivery.

### Study and Data Integrity

The study design will be guided by the CONSORT (Consolidated Standards of Reporting Trials) statement [31]. Randomisation will occur using a computer-generated random number sequence undertaken by the study manager and concealed from investigators. The intervention protocol will be detailed in a study manual, and a minimum of 20% of intervention sessions will be reviewed by an external reviewer to ensure adherence to the delivery of the intervention protocol. Self-report measures are commonly used to assess sun-exposure although they may be subject to bias [32], therefore a sub-sample (n = 40) of participants will wear polysulphone (sun) badges over a two day period to check the reliability of the self-reported sun exposure data.

### Measurement

Data will be collected by self-reported pre- and post-intervention questionnaires. The pre-intervention questionnaire will be completed in the class room one week before the commencement of the intervention. The post-intervention questionnaires will be completed at one week and four weeks after the commencement of the intervention.

#### Variables

Demographic data collected pre-intervention will include age (in years) and sex (male or female). Data will also be collected on colour of skin before tanning (very fair, fair, olive or brown, Asian, black), colour of skin with repeated exposure to the sun light without protection (go very brown and deeply tanned, get moderately tanned, get mildly or occasionally tanned, get no suntan at all or occasionally get freckled), natural hair colour (black, dark brown, light brown, dark blonde, light blonde, red), eye colour (dark brown, light brown, green, blue), and hours spent in the sun in the past week.

Primary outcome variables will assess the effectiveness of the intervention in improving students' sun protection intentions and behaviour. Secondary outcome variables will assess the effectiveness of the intervention as a means of improving students' attitudes toward performing sun protective behaviours (i.e., attitudes), perceptions of normative support to sun protect (i.e., subjective norms, group norms, and image norms), and perceived control over performing sun protective behaviours (i.e., perceived behavioural control) (Table 1).

**Table 1:** Primary and Secondary Outcome Measures

Domain	Variable	Number of items	SCALE	MEASUREMENT STRATEGIES
**Primary outcome variables**				

	Intention	4	1 (strongly disagree) to 7 (strongly agree)	"I am willing to perform sun-protective behaviours."; "I intend to perform sun-protective behaviours."; "I plan to perform sun-protective behaviours."; "It is likely that I will perform sun-protective behaviours."

	Behaviour	3	1 (never) to 7 (always)	"Think about the past week. In general, how often did you perform sun-protective behaviour?" "Think about the past week, how often did you perform sun-protective behaviour on a school day?"; "Think about the past week, how often did you perform sun-protective behaviour on the weekend?"

**Secondary outcome variables**				

	Attitude	6	1 (pleasant) to 7 (unpleasant) 1 (good) to 7 (bad) 1 (wise to 7 (unwise) 1 (difficult) easy (7) 1 (nice) to 7 (awful) 1 (negative) to 7 (positive)	"Performing sun-protective behaviours every time I go in the sun for more than 10 minutes during the next week, would be..."

	Subjective Norms	2	1 (strongly disagree) to 7 (strongly agree)	"Those people who are important to me would want me to perform sun-protective behaviours." and "Most people who are important to me would approve of me performing sun-protective behaviours."

	Perceived Behavioural Control	4	1 (strongly disagree) to 7 (strongly agree)	"I have complete control over whether I perform sun-protective behaviours."; "It is mostly up to me whether I perform sun-protective behaviours."; "If I wanted to it would be easy for me to perform sun-protective behaviours."; "I am confident that I could perform sun-protective behaviours."

	Group Norms	4	1 (strongly disagree) to 7 (strongly agree) 1 (none) to 7 (all)	"Most of my friends perform sun-protective behaviours." and "My friends think that performing sun-protective behaviours is a good thing to do." "How many of your friends would think that performing sun-protective behaviours every time you are out in the sun for more than 10 minutes in the next week is a good thing to do?" and "How many of your friends would perform sun-protective behaviours every time they are out in the sun for more than 10 minutes during the next week?"

	Image Norms	5	1 (strongly disagree) to 7 (strongly agree)	"Young celebrities and movie stars always seem to have a tan."; "I see more examples of models who have pale skin on TV and in magazines than I used to."; "I think that to be a successful movie star or TV star you should have a tan."; "It seems that society wants young people to have a tan."; "I can think of many young movie stars and TV stars who have pale skin."

The target behaviour is "Performing sun-protective behaviours (i.e., using SPF 30+sunscreen, wearing protective clothing such as a hat, long-sleeved shirt and sunglasses, and seeking shade between 10 am and 3 pm) every time you go in the sun for more than 10 minutes during the next week". To maximise congruence between the prediction and criterion variables, the TPB variables (i.e., intention, attitude, subjective norms, perceived behavioural control) are measured at the same level of specificity in terms of action, target, and time [15]. The items are constructed in strict accordance with TPB recommendations [15] and are each scored on a 7-point Likert scale, except for attitude, which is scored on 7-point semantic-differential scales.

#### Intervention Implementation

At the conclusion of each session, participants and facilitators will evaluate the program content, materials, and delivery. Adherence to the program will be assessed and recorded after each intervention session by the project manager, including information on student participation, the completion of all parts of the individual activities, and the achievement of the aims of each of the sessions.

### Data Analyses

Chi square (categorical variables), ANOVA (normally distributed continuous variables), and Kruskal-Wallis tests (non-parametric variables) will be used to compare baseline characteristics between groups, as well as between those with complete data and those who withdrew or were lost to follow-up. Outcomes will be analysed using general linear models for each of the change outcomes, including the main effects of group and time and the interaction of group and time. Sensitivity analyses will be conducted to determine the effect of missing data. All data analyses will be conducted on the basis of intention to treat principles [33].

## Discussion

This study trials a school-based intervention to promote sun protective behaviour in adolescents. To date, few studies have focused on understanding the psychosocial processes surrounding sun protection in an Australian context, and there is an associated absence of theory-based interventions to address these. This theory-driven multi-component intervention accounts for the range of psychosocial factors impacting upon Australian adolescents' sun safe decisions and, if effective, will contribute to increased sun protective behaviour that is crucial for reducing the incidence of skin cancer and the resulting burden of disease. The intervention will be immediately translatable into practice by trained staff and may be tailored to suit other high-risk groups.

## List of abbreviations used

SPF: Sun protection factor; TPB: Theory of planned behavior; ANOVA: Analysis of variance

## Competing interests

The authors declare that they have no competing interests.

## Authors' contributions

KMW, ALH and RMcDY conceptualised the study. All authors further developed the study protocol and are responsible for the implementation of the intervention. ALH was responsible for drafting the manuscript and all authors contributed to the revision of the manuscript and accept responsibility for and approve of the final version.

## Pre-publication history

The pre-publication history for this paper can be accessed here:

http://www.biomedcentral.com/1471-2407/12/1/prepub
